# Editorial: Synaptic Loss and Neurodegeneration

**DOI:** 10.3389/fncel.2021.681029

**Published:** 2021-04-21

**Authors:** Jaichandar Subramanian, Marie-Ève Tremblay

**Affiliations:** ^1^Department of Pharmacology & Toxicology, University of Kansas, Lawrence, KS, United States; ^2^Axe Neurosciences, Centre de Recherche du CHU de Québec, Université Laval, Québec City, QC, Canada; ^3^Neurology and Neurosurgery Department, McGill University, Montréal, QC, Canada; ^4^Department of Molecular Medicine, Université Laval, Québec City, QC, Canada; ^5^Division of Medical Sciences, University of Victoria, Victoria, BC, Canada; ^6^Department of Biochemistry and Molecular Biology, The University of British Columbia, Vancouver, BC, Canada

**Keywords:** synaptic loss, neurodegenerative diseases, glial cells, astrocytes, microglia, multiphoton imaging, periphery, gut microbiome

Synapse loss is associated with sensory, motor, and cognitive impairments in a variety of neurodegenerative conditions, such as major depressive disorder, schizophrenia, Alzheimer's disease, Huntington's disease, and amyotrophic lateral sclerosis, as well as aging. Loss of excitatory synapses is the strongest correlate for cognitive impairments in Alzheimer's disease. Despite the overwhelming evidence for synapse loss in contributing to disease etiology, very little is known about the mechanisms involved. Introducing disease linked mutations in model organisms has provided an entry point to address the mechanisms of synaptic dysfunction. Though this has led to many exciting discoveries, our current understanding has not reached the threshold required to develop translational approaches to treat synaptic abnormalities. To shine some light on this very important topic, this Research Topic provides a collection of primary research articles, reviews as well as mini reviews, and a perspective article, on different aspects of synaptic dysfunction in the context of neurodegenerative diseases.

The emerging roles of microglia and astrocytes in synaptic loss are first discussed in a series of reviews and mini-reviews. Lee and Chung cover the recent developments on the physiological roles of glial cells at synapses, and propose that synaptic loss can be initiated by a misregulation of normal glial functions. Henstridge et al. (2016) next synthesizes the recent literature on the contribution of glial cells to excitatory/inhibitory imbalance, in the context of prevalent neurodegenerative diseases: Alzheimer's disease, Parkinson's disease, amyotrophic lateral sclerosis, and multiple sclerosis. Focusing on Alzheimer's disease, Ziegler-Waldkirch and Meyer-Luehmann further summarize the roles of microglia and astrocytes in synaptic loss in genetic mouse models, and discuss the current therapeutic avenues. Pons and Rivest also cover important therapeutic avenues of macrophage colony-stimulating factor and its receptor expressed by peripheral monocytes, macrophages and microglia, in Alzheimer's disease, multiple sclerosis, glioma, and brain injury.

In complement, Subramanian et al. review the role of cell-intrinsic calcium dyshomeostasis and cell-extrinsic activities of microglia in the mechanisms underlying synaptic loss in genetic mouse models of Alzheimer's disease. Chang et al. further discuss the involvement of neuronal hyperpolarization-activated cyclic nucleotide-gated channels in major depressive disorder, Parkinson's disease, Alzheimer's disease, amyotrophic lateral sclerosis, and spinal muscular atrophy. Pradhan et al. summarize the evidence for an altered brain derived neurotrophic signaling in amyotrophic lateral sclerosis and provide insights into its modulation as a neuroprotective strategy. Liu et al. additionally expose the current state of knowledge on the pathophysiological roles of neuron- and glial-derived exosomes in different diseases of the central nervous system and their application as non-invasive biomarkers in the cerebrospinal fluid and peripheral body fluids. In a perspective article, Zhao et al. discuss the interaction between the periphery and the central nervous system, particularly focused on the gut microbiome and its modulation of neurofilaments and synaptic signaling proteins upon bacterial infection.

A series of primary research articles also unravel mechanisms of synaptic loss in different pathological conditions using different model organisms, from drosophila to rodents. In particular, Chen et al. reveal an altered synaptic vesicle release and calcium influx at single presynaptic terminals of cortical neurons in a knockin mouse model of Huntington's disease. Spring et al. provide results from fourteen different drosophila lines expressing spinal muscular atrophy patient-derived mutations, showing a comprehensive modeling of the disease features including locomotor decline. Kriebel et al. propose a novel model of age-related decline in synaptic connectivity, involving a partial inhibition of the mitochondrial respiratory chain, *in vitro* and *in vivo* in rats. Lastly, Hui et al. (2018) uncover partial sex differences in microglial phagocytic activity, excitatory, and inhibitory synaptic density, as well as inhibitory synaptic tone and excitatory synaptic transmission in a mouse model of maternal immune activation.

In conclusion, this special issue provides different perspectives on the neuronal, glial, and peripheral mechanisms involved in synaptic loss across a wide range of neurodegenerative disease conditions, including major depressive disorder, Parkinson's disease, Alzheimer's disease, amyotrophic lateral sclerosis, multiple sclerosis, spinal muscular atrophy, glioma, and brain injury. Novel model organisms also are proposed and shown to provide insights into the mechanisms at play in Huntington's disease, spinal muscular atrophy, cognitive aging, and maternal immune activation, which is associated with the onset of various neuropsychiatric disorders and neurodegenerative diseases in human.

## Author Contributions

JS and M-ÈT wrote the manuscript. Both authors contributed to the article and approved the submitted version.

## Conflict of Interest

The authors declare that the research was conducted in the absence of any commercial or financial relationships that could be construed as a potential conflict of interest.
